# Breastfeeding and Weaning: Practices in Urban Slums of Southern Punjab, Pakistan

**DOI:** 10.7759/cureus.2189

**Published:** 2018-02-13

**Authors:** Khalil Ahmed, Muhammad Talha, Zainab Khalid, Mehvish Khurshid, Rizwan Ishtiaq

**Affiliations:** 1 Department of Medicine, Hull Royal Infirmary; 2 Medicine, Bahawal Victoria Hospital, Quaid-E-Azam Medical College, Bahawalpur; 3 Department of Medicine, Rawalpindi Medical College, Rawalpindi, Pakistan; 4 Department of Medicine, Bahawal; 5 Gastroenterology, Beth Israel Deaconess Medical Center, Boston

**Keywords:** breastfeeding, weaning, infants, mother

## Abstract

Objective

Proper breastfeeding and weaning practices are effective ways of reducing childhood morbidity and mortality. This study aimed to find out breastfeeding and weaning practices among infants of six months to one year in urban slums of Bahawalpur city. By evaluating the knowledge and attitude of lactating mothers regarding their child feeding habits, modifications and specific interventions can be implemented to improve the breastfeeding and weaning practices of the area.

Methods

A cross-sectional descriptive study was carried out in the Shahdrah slums of Bahawalpur City after getting approval from the institutional review board of Quaid-e-Azam Medical College, Bahawalpur. The survey was conducted from April 10, 2017 to May 30, 2017. One hundred mothers of infants aging six months to one year of age were interviewed. Mothers filled a customized questionnaire, consisting of questions about logistic variables, breastfeeding, and weaning. Statistical analysis was performed using Statistical Package for the Social Sciences (SPSS) version 22.0 (IBM Corp. Armonk, NY, USA).

Results

The mean age of the women was 24+2 years. It was found that 74% (n=74) of the mothers had one to three children, 85% (n=85) of the mothers were breastfeeding their infants at the time of the study, 40% (n=40) of the mothers were illiterate, 16% (n=16) of the mothers had secondary education, and 87% (n=87) of the mothers were nonworking women. Out of 85 women who were breastfeeding, 95% (n=80) of the women were 18-23 years of age. We found that 57% (n=57) of the infants were six to nine months old and the remaining 43% (n=43) were up to 12 months of age. Regarding the initiation of breastfeeding, 60% of the females started soon after delivery, and 32% started within two to seven days. We found that 70% (9/13) of the working women and 87.4% (76/87) of the non-working women were breastfeeding. The study found that 92.3% (12/13) of the working women and 88.5% (77/87) of the non-working women were weaning.

Conclusion

A majority of the mothers were breastfeeding and weaning their babies. The factors documented were young age, low parity, no working status, and nuclear families. The commonly used feeding materials were cereals, banana, rice, and bread. No side effects of weaning were observed. Weaning was associated by late age, parity, non-working status, educational status of father, and nuclear families.

## Introduction

Breastfeeding is the feeding of an infant or a young child with breast milk, and it remains the purest, healthiest, and least expensive feeding method that fulfills their needs. The gradual introduction of semi-solid foods, known as the weaning process, is essential to meet the increased nutritional requirement during an infant’s first year. Weaning should be started after the age of six months and should contain energy-rich semi-solid foods.

According to the World Health Organization (WHO), it is recommended to feed infants exclusively with breast milk for the first six months followed by the addition of compatible foods along with breast milk after six months up to two years of age [1]. The beneficial effects of breastfeeding depend on breastfeeding initiation, its duration, and the age at which the breastfed child is weaned. Breastfeeding is the best way of providing the ideal food for healthy growth and development of infants, and its advantages range from physiological to psychological for both mothers and infants. Breastfeeding is a natural source of child spacing, and it reduces the chances for breast cancer to develop in females [2]. It is an excellent nutritional source for the baby, reducing the incidence of diseases by enhancing the immunity and decreasing chances to develop allergies, obesity, or diabetes in the baby. This leads to reduced rates of hospitalizations. Psychologically, it increases the infant-maternal bond and is a source of great satisfaction for the mother. During the first half hour after birth, the newborn is very active. Newborns can be fed with breastmilk within four to six hours after cesarian deliveries [3]. There are several factors that have been reported to affect breastfeeding of the baby. Some of these factors include soreness of nipples and the perception of the mother that she is not producing an adequate amount of milk. Employment and length of maternity lead to insufficient awareness regarding the benefits of breastfeeding. Other factors include lack of knowledge by health care professionals, cultural beliefs regarding unhygienic practices like pre-lacteal feeds (e.g., honey), and lack of guidance from peers and families [4,5].

The gradual introduction of semisolid foods, known as the weaning process, is essential to meet the increased nutritional requirement during an infant’s first year. Weaning should be started after the age of six months and should contain energy-rich semisolid food [6]. Most of the mothers either initiate early or delayed weaning, which has a harmful effect on the growth and development of the child. Improper execution of weaning makes a child susceptible to malnutrition and diarrhea [7]. Common food items given during weaning are bananas, daliya (porridge), khichri (special Indian rice recipe), yogurt, and cereals. Feeding practices during infancy are essential determinants of future physical and mental well-being because of the rapid growth spurt and development of organs and tissues during the first year of life. If the weaning is at the proper time, the child gains weight and is healthy. It is necessary to take care of hygiene to avoid problems during weaning. Milk feeding must be carried out along with weaning. Delayed weaning can lead to serious health complications for infants because, after six months of age, breast milk alone is not sufficient both in quality and quantity to meet the nutritional requirement of the child, especially for energy and micronutrients [8]. Breast milk is notably insufficient in vitamin A, iron, and zinc requirements of a nursing baby.

This study was done to assess the breastfeeding and weaning practices among mothers of infants in urban slums of Bahawalpur so that a general trend can be found and strategies can be implemented. Based on our survey, we have analyzed several factors associated with breastfeeding and weaning and how they affect such practices in daily life.

## Materials and methods

This was a cross-sectional descriptive study carried out in the Shahdrah slums of Bahawalpur City. The study was conducted from April 10, 2017 to May 30, 2017. One hundred mothers of infants aged six months to one year of age were interviewed. Informed consent was taken from all the participants before participating in the study. Nonprobability convenient sampling technique was used. Data was collected using preformed, pretested questionnaires that comprised two parts. The first part included demographic variables such as age, education, and living children. The second part consisted of variables like breastfeeding and weaning practices. The data was analyzed using Statistical Package for the Social Sciences (SPSS) version 22.0. (IBM Corp. Armonk, NY, USA). The mean and the standard deviation were calculated for numerical data like age. Frequencies and percentages were calculated. Stratification was done according to age, women’s education level, husband’s education level, the occupation of women, type of family, and the number of living children. Chi-square test was applied to see if any statistical difference between groups existed. P value ≤ 0.05 was taken as significant.

## Results

The mean age of the 100 women was 24+2 years. Based on age, mothers were divided into five groups as shown in the Table 1.

**Table 1:** Stratification of women based on age and breastfeeding habits

Age of Mother in Years	Breastfeeding				Total
	No (Frequency)	Percentage	Yes (Frequency)	Percentage	
18-23	1	4.3%	22	95.7%	23
24-29	10	23.8%	32	76.2%	42
30-35	4	12.5%	28	87.5%	32
36-41	0	0%	1	100%	1
42-47	0	0%	2	100%	2

We found that 74% (n=74) of the mothers had one to three children, 21% (n=21) had four to six children, and 5% (n=5) had seven to nine children each (Table 2).

**Table 2:** Stratification of women based on number of children and breastfeeding habits

No of Children	Breastfeeding				Total
	No (Frequency)	Percentage	Yes (Frequency)	Percentage	
1-3	12	16.2%	62	83.7%	74
4-6	2	9.5%	19	90.4%	21
7-9	1	20%	4	80%	5

With regard to the educational status of mothers, the study found that 40% (n=40) of the mothers were illiterate, 17% (n=17) had primary level education, and 16% (n=16) had above secondary level education (Table 3).

**Table 3:** Stratification of women based on educational status and breastfeeding habits

Educational Status of the Mothers	Breastfeeding				Total
	No (Frequency)	Percentage	Yes (Frequency)	Percentage	
Illiterate	6	15%	34	85%	40
Primary level	2	11.7%	15	88.2%	17
Middle level	0	0%	13	100%	13
Secondary Level	4	28.5%	10	71.5%	14
Above secondary level	3	18.7%	13	81.3%	16

Stratification of the mothers based on working status and breastfeeding is illustrated in Table 4.

**Table 4:** Stratification of women based on work status and breastfeeding habits

Working Status	Breastfeeding				Total
	No (Frequency)	Percentage	Yes (Frequency)	Percentage	
Working	4	30.7%	9	69.3%	13
Non-working	11	12.6%	76	87.4%	87

With regard to the age of the infants, the study found that 57% (n=57) of the infants were of six to nine months of age and the remaining 43% (n=43) were up to 12 months old (Table 5).

**Table 5:** Stratification of infants based on age and breastfeeding habits

Age of Infants (in Months)	Breastfeeding				Total
	No (Frequency)	Percentage	Yes (Frequency)	Percentage	
6-9 months	10	17.5%	47	82.5%	57
Up to 12 months of age	5	11.6%	38	88.4%	43

The gender distribution of the infants was as follows: 60% (n=60) were male while 40% (n=40) were female (Table 6).

**Table 6:** Gender of infants and breastfeeding habits

Gender of Baby	Breastfeeding				Total
	No (Frequency)	Percentage	Yes (Frequency)	Percentage	
Male	9	15%	51	85%	60
Female	6	15%	34	85%	40

When we analyzed the gender of the infants and breastfeeding practices, we found that both male and female infants were equally breastfed (85%). Families were divided into three groups, i.e., nuclear, extended, and polygamous (Table 7).

**Table 7:** Type of family respondents and breastfeeding habits

Type of Family	Breastfeeding				Total
	No (Frequency)	Percentage	Yes (Frequency)	Percentage	
Nuclear	7	22.5%	24	77.5%	31
Extended	7	13%	47	87%	54
Polygamous	1	6.7%	14	93.3%	15

Regarding the initiation of breastfeeding, 60% of the females started soon after delivery, 32% within two to seven days, while 10% began within 24 hours of birth. Regarding pre-lacteal feeding practices among mothers, 72% gave *ghutti* while 28% did not. The frequency of different food items given in *ghutti* showed 42% honey, 9% dates and zam-zam, and in 40% of the cases, other things were given as ghutti. Regarding the colostrum feeding practices, 52% of the mothers gave colostrum to their babies while 48% did not. Regarding the frequency of breastfeeding, 80% of the mothers breastfed their babies on demand while 20% breastfed on a fixed schedule. According to the study, 51% of the mothers were not exclusively breastfeeding their baby, while 49% of the mothers were. Regarding the reasons for not breastfeeding, the majority (47%) considered other milk products more beneficial, 32% did not feed their baby due to pressure/fear, while 7% thought it was embarrassing, old-fashioned, and that it prevents them from going to their jobs. Regarding the weaning practices among mothers, 90% had started weaning while 10% had not started yet. Regarding the time of initiation of weaning, 29% of mothers began at six months or after six months, while 24% started between four to six months and 18% at four months. The food items used for weaning by the mothers were cereals (15.4%), banana (13.8%), khichdi (12.6%), rice (12.2%), bread (11.7%), biscuits (11.6%), porridge (11.4%), and juices (11.1%). Regarding the frequency of weaning practices among infants, the study revealed that a majority (42%) of the infants were fed two to three times, 34% were fed more than three times, and 24% once a day. Among the side effects of weaning observed by mothers, the majority (73%) saw no side effect while 18% observed diarrhea, 6% observed vomiting, and 3% observed fever.

The relation between the age of the mothers and weaning practice is illustrated in Table 8.

**Table 8:** Stratification of women based on age and weaning practices

Age of Mother in Years	Weaning				Total
	No (Frequency)	Percentage	Yes (Frequency)	Percentage	
18-23	2	8.7%	21	91.3%	23
24-29	6	14.2%	36	85.8%	42
30-35	2	6.25%	30	93.75%	32
36-41	0	0%	1	100%	1
42-47	1	50%	1	50%	2

With regard to the number of children the mothers had and weaning practices, we found that all women having seven to nine children started weaning 100%, while mothers with one to three children were practicing 90.5%, and 81% of the mothers with four to six children were practicing (Table 9).

**Table 9:** Stratification of women based on number of children and weaning practices

No of Children	Weaning				Total
	No (Frequency)	Percentage	Yes (Frequency)	Percentage	
1-3	7	9.5%	67	90.5%	74
4-6	4	19%	17	81%	21
7-9	0	0%	5	100%	5

In the sample of 100 mothers, the relation between the educational status of the mothers and weaning practices showed that weaning is 100% in the mothers with secondary education, while 87.5%, 83.4%, 92.3%, and 85.7% belonged to illiterate, up to primary, up to middle, and secondary educated, respectively (Table 10).

**Table 10:** Stratification of women based on educational status and weaning practices

Educational Status of the Mothers	Weaning				Total
	No (Frequency)	Percentage	Yes (Frequency)	Percentage	
Illiterate	5	12.5%	35	87.5%	40
Primary level	3	17.6%	14	82.4%	17
Middle level	1	7.7%	12	92.3%	13
Secondary Level	2	14.3%	12	85.7%	14
Above secondary level	0	0%	16	100%	16

The relation between the working status of the mother and weaning practices is as follows: it is 92.3% in working mothers and 85.5% in non-working mothers (Table 11).

**Table 11:** Stratification of women based on work status and weaning practices

Working Status	Weaning				Total
	No (Frequency)	Percentage	Yes (Frequency)	Percentage	
Working	1	7.7%	12	92.3%	13
Non-working	10	11.5%	77	88.5%	87

When we looked at the age of infants and weaning practices, we found that 86% of infants started to wean during six to nine months of age while 93% started to wean during ten to twelve months of age (Table 12).

**Table 12:** Stratification of infants based on age and weaning habits

Age of Infants (in Months)	Weaning				Total
	No (Frequency)	Percentage	Yes (Frequency)	Percentage	
6-9 months	8	14%	49	86%	57
Up to 12 months of age	3	7%	40	93%	43

Our study found that 85% of male babies were weaned as compared to 95% of female babies (Table 13).

**Table 13:** Gender of infants and weaning habits

Gender of Baby	Weaning				Total
	No (Frequency)	Percentage	Yes (Frequency)	Percentage	
Male	9	15%	51	85%	60
Female	2	5%	38	95%	40

The relation between the type of family and weaning practice was as follows: 93.5% in nuclear, 93.3% in polygamous, and 85.2% in extended families (Table 14).

**Table 14:** Type of family respondents and weaning practices

Type of Family	Weaning				Total
	No (Frequency)	Percentage	Yes (Frequency)	Percentage	
Nuclear	2	6.5%	29	93.5%	31
Extended	8	14.8%	46	85.2%	54
Polygamous	1	6.7%	14	93.3%	15

## Discussion

Breastfeeding is the most convenient mode of feeding an infant. It is free of cost and contamination, fresh and ideal regarding nutrition in the initial year of life. In some parts of our society, breastfeeding is considered a backward form of feeding a baby in comparison to bottle feeding, which is deemed to be sophisticated. Breastfeeding also prevents breast engorgement. In December 2013, the Department of Pediatrics at Quaid-e-Azam International Hospital Islamabad, (Pakistan) researched the reasons of failure of breastfeeding in children less than six months of age [9]. Amongst a total of 300 mothers, 135 (43.5%) were illiterate, 94 mothers (30.3%) were educated up to secondary school, and only 13 mothers (4.2%) had completed graduation. The study found that 289 mothers (91%) were housewives, and just 28 mothers (9%) were working women. The age of the infants ranged from 7-180 days with the mean of 99.2+57.9 days. In the study, 185 infants were male, and 125 infants were female (59.71% vs. 40.3%). Two babies (0.6%) were breastfed within the first hour after birth. Breastfeeding was started within one to six hours and after six hours in 153 (49.4%) and 155 (50%) infants, respectively. Insufficient milk production was reported to be the most common cause of failure to breastfeed exclusively (93.2%). Other reasons included the working status of the mother (4.2%), illness of baby (0.65%) or disease of the mother (1.9%).

A study conducted in Mauritius assessed breastfeeding and infant feeding patterns among 500 mothers [1]. Most of the participants had an age range between 25-31 years, and most of them were married. Sixty percent of the participants started breastfeeding their babies the same day after delivery and the remaining 40% started nursing their baby after 24 hours of delivery. Only 18% of the mothers exclusively breastfed their infants for the first six months. During weaning, feeding materials included homemade and commercially available food products.

In another study in NoorPur Shahan, Islamabad, Pakistan, 138 local mothers were questioned regarding their breastfeeding and weaning practices [10]. A significant portion of the sample had an age range of 23-26 years. In this study 93.4% of the respondents revealed that breastfeeding is beneficial for the newborns. Thirty-three percent mothers breastfed their infants for up to six months and discontinued afterward. Fifty-two percent of the mothers breastfed their newborns for six months to 30 months. Almost 58% of the mothers reported liquids or water to be unsafe for newborns.

According to a prospective observational study of 600 mothers carried out in Southern Vietnam, 93.2% women were found literate amongst the total of 600 [11]. In this study 98.5% women reported that they have a stable family and living. This is also an essential factor for the health of the child. Sixty-nine percent of the total births took place in hospital settings, 27% in community health settings, and 2.7% in domestic settings. Eighty-nine percent of the mothers breastfed their infants exclusively up to six months. Ninety-four percent of the mothers had awareness regarding neonatal tetanus, 87.5% had awareness regarding hypothermia, and 61.3% of the mothers knew how to deal with diarrhea.

Our survey showed different results than those found in the study from Mauritius [1]. Most of the women included in our research were housewives, so more breastfeeding practice was observed, and there is more emotional attachment between mothers and their babies. Our religious and cultural values also promote breastfeeding. More than half of the women in our research started breastfeeding soon after delivery. Similar results were found in the research carried out in Mauritius [1]. This contrasted with results of research carried out in Islamabad [9]. The deliveries of most of the women in our research were handled by lady health workers, so breastfeeding was started soon after delivery. While in C-section, breastfeeding cannot be quickly started due to general anesthesia [6]. Our research revealed about half of the women giving colostrum to their babies. This is comparable to the findings of a study conducted in India where three-fourths of the women gave colostrum to their babies.

According to our research, most mothers breastfed their babies on demand. Half of the mothers were exclusively breastfeeding their babies. Similar results were found in a study carried out in the Southern province of Vietnam [11]. Contrasting results were found in Mauritius and NoorPur Shanan where exclusive breastfeeding was much less practiced [1,10]. Most of the mothers included in our research were housewives, so they had more time to feed their babies. They were more conscious about the health of their babies, so they fed them on demand. In our research, few women did not feed their babies due to pressure and fear while a few considered it embarrassing, old-fashioned, and a hindrance to going for a job. Moreover, most of the women included in our research were housewives, and they followed cultural and religious values. Most infants were breastfed up to 12 months of age. A period of 12 months was considered ideal for breastfeeding as the infant has met development milestones. We found in our study that both male and female infants were equally breast-fed, in contrast with the result found in a cross-sectional study carried out in East Africa in which the duration of breastfeeding was more for males as compared to female infants [12]. The women included in our research were less biased. They followed religious values and gender equality. In our research, breastfeeding among infants belonging to polygamous families was much more as compared to that in nuclear or extended families. The workload was divided among women of polygamous families, so they had more time to breastfeed their babies.

Regarding the frequency of weaning practices among infants, our research revealed that a majority of women fed their babies two to three times a day (42%). One-fourth of the women did not continue breastfeeding with weaning while the rest of the 75% continued. In our research, there was a more emotional attachment between mothers and their infants. Mothers were more conscious about the health of babies, so they continued breastfeeding with weaning for better health of babies. Many of the women in our research observed no side-effects of weaning. Mothers used fresh, nutrition-rich homemade food items for weaning their babies. In our research, the weaning practice was more among working women and men as compared to that in non-working women and men (92.3% vs. 88.5%). As most of the mothers included in our research were housewives and fathers were economically productive, the weaning practice was high among them. Regarding the gender of the infants, the results of our research revealed more weaning practice among female babies. Female babies included in our research grew weak due to avoidance by the father and other family members, so to compensate for this, they were weaned more. Moreover, owing to family and cultural pressure, the next pregnancy occurred earlier after the mother gave birth to a female baby, so cessation of breastfeeding and the start of weaning occurred earlier among these women.

## Conclusions

Most of the women in our study breastfed their infants. The factors responsible were young age, low parity, no working status, and nuclear families. Most of the women weaned their infants. Common food items used for weaning were cereals, banana, khichri, rice, and bread. Most of the women experienced no side effects. Weaning was associated with late age, low parity, non-working status, and extended families. Breastfeeding is a crucial child survival strategy. Breast milk meets all the nutritional needs of the baby safely and adequately. In developing countries like Pakistan where malnutrition is common and infant mortality rate is high, all possible measures should be taken to support and promote breastfeeding. Strategies aimed at fostering breastfeeding and weaning practices must be developed. Periodic surveys should be done to assess the determinants of breastfeeding and weaning practices. Awareness campaigns should be designed. Training programs regarding breastfeeding should be prepared for implementation by obstetrics and pediatrics residents with the help of the nursing staff.
